# How School Climate Influences Teachers’ Emotional Exhaustion: The Mediating Role of Emotional Labor

**DOI:** 10.3390/ijerph121012505

**Published:** 2015-10-08

**Authors:** Xiuping Yao, Meilin Yao, Xiaoli Zong, Yulan Li, Xiying Li, Fangfang Guo, Guanyu Cui

**Affiliations:** 1 School of Psychology, Beijing Normal University, Beijing 100875, China; E-Mails: xiupingyao@mail.bnu.edu.cn (X.Y.); psy_zongxiaoli@163.com (X.Z.); lanliyu311@163.com (Y.L.); xchcgy@126.com (G.C.); 2 MOE Key Laboratory of Modern Teaching Technology, Shaanxi Normal University, Xi’an 710119, China; E-Mail: flylee007@163.com; 3 School-based Mental Health Center, Beijing Information Science and Technology University, Beijing 100192, China; E-Mail: guofangfang@mail.bnu.edu.cn

**Keywords:** school climate, emotional labor, surface action, deep action, emotional exhaustion, teacher

## Abstract

Currently, in China, improving the quality of teachers’ emotional labor has become an urgent need for most pre-kindergarten through 12th grade (p–12) schools because the new curriculum reform highlights the role of emotion in teaching. A total of 703 primary and high school teachers in Mainland China were investigated regarding their perceptions of school climate, emotional labor strategy and emotional exhaustion via questionnaires. The findings revealed that the teachers’ perceptions of the school climate negatively affected surface acting but positively affected deep acting. Surface acting positively predicted emotional exhaustion, and deep acting had no significant effect on emotional exhaustion. Moreover, emotional labor mediated the relationship between the teachers’ perceptions of the school climate and emotional exhaustion. Programs aimed at improving the school climate and the teachers’ use of appropriate emotional labor strategies should be implemented in schools in Mainland China.

## 1. Introduction

Teaching is an emotionally demanding profession and may require a teacher to devote his or her whole heart to facilitating students’ development [1,2,3]. In pre-kindergarten through 12th grade (p–12 ) schools, teachers are expected to meet the diverse needs of students and address various misbehaviors on a daily basis. Moreover, to create a warm and safe classroom climate, teachers have to display positive emotions and hide negative emotions. In short, teachers engage in emotional labor. 

The term emotional labor was created by Hochschild in 1983 and refers to managing one’s emotions to comply with organizational or occupational display rules [4]. Presently, many organizations are focusing more attention on the role of emotional labor in job performance [5,6]. For teachers, emotional labor may positively affect teacher-student relationships, which are an important contributor to teachers’ well-being [7,8]. However, emotional labor can also be harmful when there is a gap between the emotions that the teachers experience and the emotions that they are required to display. This gap has been labeled emotional dissonance or emotion-rule dissonance [9]. Emotional dissonance can lead to increased stress, which in turn may drive teachers to reduce their feelings of discomfort by regulating their emotions [10]. There are two main emotional regulation strategies, surface acting and deep acting. During surface acting, people hide their authentic feelings or express fake emotions to meet display rules [9]. For example, a teacher may suppress negative emotions (e.g., disappointment, anger and disgust) when interacting with students or parents. During deep acting, people attempt to actually experience the required emotions [9]. For example, a teacher who feels angry about the misbehavior of a student may attempt to reappraise the behavior to alleviate their anger and to display positive emotions to the students. According to ego depletion theory, both surface and deep acting require mental energy and resources; thus, both strategies are associated with emotional exhaustion [10]. Previous studies have consistently shown that surface acting is positively related to emotional exhaustion among Western [5,11,12] and Chinese samples [13]. However, the relationship between deep acting and emotional exhaustion seems less clear across cultures. Deep acting is not related to burnout among Chinese primary and junior high school teachers [13], but it is negatively related to emotional exhaustion among Turkish primary school teachers [14]. Additionally, research has revealed that deep acting is negatively related to emotional exhaustion among Chinese kindergarten teachers [15], but the same result was not observed among Chinese primary and secondary school teachers [13]. Thus, it is necessary to clarify the effects of deep and surface acting on emotional exhaustion in different cultural contexts and to reveal the underlying mechanism.

To further explore the process and mechanism of emotional labor, it is important to understand the factors that influence teachers’ uses of certain strategies. Unfortunately, little research has focused on the antecedents of emotional labor [16]. According to affective event theory (AET), the work environment (e.g., organizational support) not only influences one’s affective states but also influences affect-driven behavioral reactions (e.g., emotion regulation strategies) [17]. Therefore, we hypothesized that the organizational environment may be a strong predictor of emotional labor. For most teachers in p–12 schools, the school climate is a highly influential environment. The school climate is widely defined as the psychosocial context within which teachers work. The school climate is a complex construct that includes such elements as the teachers’ perceptions of cooperation among colleagues, students’ motivation and behavior, school’s resources and innovation, and decision-making authority [18]. A high quality school climate can elicit a teacher’s sense of obligation to repay the organization. Based on social exchange theory [19], when an employee perceives that the organization values his/her contributions and well-being and provides timely and appropriate support whenever needed, the employee will feel obliged to repay the organization. Similarly, teachers with perceptions of high-quality school climates may devote greater amounts of cognitive, emotional and behavioral resources to their work to help the school achieve its goals while simultaneously attempting to actually experience the prescribed emotions. In other words, these teachers tend to perform deep acting and regulate their inner feelings according to the display rules of the school. In contrast, teachers with perceptions of a negative school climate may not feel an obligation to repay the school. These teachers are less likely to engage in activities that are beneficial to the school and put less effort into displaying the expected emotions; that is, they tend to perform surface acting without regulating their inner feelings. 

Previous studies have shown that teachers’ perceptions of the school climate contribute to a series of teacher outcomes, such as burnout [20,21,22], commitment [23], and job satisfaction [24,25,26,27]. Given that both the perceived school climate and emotional labor are predictors of teacher burnout and that the school climate may also influence emotional labor, it is reasonable to hypothesize that emotional labor mediates the relationship between the school climate and burnout. However, few studies have directly examined the relationships between these three variables. The purpose of the present research was to investigate the relationship between teachers’ perceptions of the school climate, emotional labor strategies and emotional exhaustion. We sought to answer the following questions: (1) Do teachers’ perceptions of the school climate affect their emotional labor strategies? (2) How do different emotional labor strategies influence emotional exhaustion among Chinese teachers? (3) Does the emotional labor strategy mediate the relationship between the perceived school climate and emotional exhaustion?

## 2. Materials and Methods 

### 2.1. Participants and Procedures

The participants were teachers from the Tianjin, Henan and Anhui provinces in Mainland China. A total of 1000 questionnaires were distributed from January to April 2014, and 703 useable questionnaires were returned, yielding a response rate of 70.3%. The participants were in-service teachers of elementary schools (226), junior high schools (261), and senior high schools (216), and 63.6% (447) of the participants were female. Regarding years of teaching experience, 20.2% (142) of the participants had below 5 years of experience, 16.9% (119) had from six to ten years, 20.1% (141) had from eleven to fifteen years, 40.7% (286) had more than fifteen years, and 2.1% (15) did not report this information. All questionnaires were completed at local teacher training classes. 

### 2.2. Ethical Principles

The present study was approved by the Research Ethics Committee of Beijing Normal University. All of the participants volunteered to participate and signed an informed consent form prior to data collection. And all data in the present study was collected anonymously. 

### 2.3. Instruments

**Teachers’ Emotional Labor Strategies**: The Teacher Emotional Labor Strategy Scale (TELSS) adapted by Yin [28] was used to measure two emotional labor strategies: surface acting (6 items, e.g., “I just pretend to have the emotions that I need to display for my job”) and deep acting (4 items, e.g., “I try to actually experience the emotions that I must show to students or their parents”). All of the items were written in Chinese and rated on a 5-point Likert scale that ranged from *strongly disagree* (1) to *strongly agree* (5). The scale has exhibited good validity and reliability in a previous study [29]. In this study, Cronbach’s α for surface acting and deep acting were 0.86 and 0.74, respectively.

**School Climate**: The School-Level Environment Questionnaire (SLEQ) revised by Johnson *et al.* [18] was used to measure the teachers’ perceptions of school climate. The scale consisted of 21 items and the following five subscales: collaboration (6 items, e.g., “Good teamwork is not emphasized enough in my school”); student relations (4 items, e.g., “Most students are helpful and cooperative with teachers”); school resources (4 items, e.g., “The supply of equipment and resources is not adequate”); decision making (3 items, e.g., “Teachers are frequently asked to participate in decisions”); and instructional innovation (4 items, e.g., “We are willing to try new teaching approaches in my school”). The items were rated on a 5-point Likert scale ranging from *strongly disagree* (1) to *strongly agree* (5). Higher scores indicated healthier school climates. The questionnaire was translated into Chinese and then back translated into English independently by two researchers. Based on an exploratory factor analysis (EFA), four items were removed and five factors were extracted from the remaining 17 items, and the factors were consistent with the five hypothesized SLEQR factors. Following the EFA, a confirmatory factor analysis (CFA) was conducted, in which the 17 SLEQ items were constrained to load on the five hypothesized factors, respectively, and the five factors loaded on an overall second-order factor, namely school climate. The results of CFA indicated that the model fit the data well: χ^2^ (110, *N* = 351) = 243.52, *p* < 0.001, root-mean-square error of approximation (RMSEA) (90% confidence interval (CI)) = 0.059 (0.049, 0.069), comparative fit index (CFI) = 0.92, Tucker-Lewis index (TLI) = 0.90, and standardized root mean square residual (SRMR) = 0.051. In the present study, Cronbach’s α coefficients for each subscale were 0.57 (Collaboration, 3 items), 0.73 (Student relations, 4 items), 0.73 (Decision making, 3 items), 0.73 (School resources, 4 items), and 0.79 (Instructional innovation, 3 items).

**Emotional Exhaustion**: The teachers’ emotional exhaustion was measured with the emotional exhaustion subscale from a modified Chinese version of the Maslach Burnout Inventory for Educators [30]. The 8 items of the subscale measured the teachers’ feeling of over-extension and tiredness (e.g., “I feel exhaustion at the end of workday”). The participants were required to respond on a 7-point Likert scale ranging from *strongly disagree* (0) to *strongly agree* (6). The scale has been shown to exhibit adequate validity and reliability in Chinese samples [30,31]. In the current study, Cronbach’s α was 0.91.

## 3. Data Analysis

Descriptive analyses were conducted to summarize the means and standard deviations of the variables. Zero-order correlations were calculated to examine the relationships between the variables. 

To address the main purpose of the study, multiple mediation models were established to explore the mediating relationships between the latent variables. According to Hu and Bentler’s suggestion [32], we used the following indices as the criteria for the model fits: RMSEA < 0.08, SRMR < 0.10, CFI > 0.90, and TLI > 0.90.

Following the procedures suggested by Preacher and Hayes [33], we tested the multiple mediation models with Mplus 7.11 to examine the total indirect effects of all of the hypothesized mediators and the specific indirect effect of each mediator. The significance of the mediating effects was examined using a bootstrapping procedure that has been recommended by many researchers [33,34,35]. The advantages of bootstrapping are not limited to its greater statistical power but also include its lack of an assumption about the normality of the distribution of the sample [36,37]. In the current study, the estimations of the parameters and confidence intervals were generated based on 5000 random samples. An indirect effect was considered significant if the 95% bias-corrected confidence interval did not include zero.

## 4. Results

### 4.1. Preliminary Analysis

The descriptive statistics and intercorrelations among the latent variables are presented in Table 1. On average, teachers’ perception of school climate was neutral (*M* (Mean) = 3.52, *SD* (Standard Deviation) = 0.62), neither very negative nor very positive (the range of the scale was from 1 to 5). Regarding emotional labor strategies, teachers reported more deep acting (*M* = 3.82, *SD* = 0.80) than surface acting (*M* = 2.03, *SD* = 0.91). Compared to the results of other two studies assessing Chinese teachers’ emotional exhaustion (*M* = 2.39, *SD* = 1.09; *M* = 2.60, *SD* = 1.03) [30,31], the level of emotional exhaustion was relatively high (*M* = 3.10, *SD* = 1.55). Moreover, all the latent variables were significantly intercorrelated in the hypothesized directions, which provided a foundation for mediation analysis. 

**Table 1:** Means, Standard Deviations and Correlations of the latent variables in this study.

Variables	M	SD	1	2	3	4
1. School climate	3.52	0.62	1			
2. Surface acting	2.03	0.91	−0.44 **	1		
3. Deep acting	3.82	0.80	0.49 **	−0.24 **	1	
4. Emotional exhaustion	3.10	1.55	−0.34 **	0.43 **	−0.11 *	1

*Note*: * *p* < 0.05, ** *p* < 0.01.

### 4.2. Measurement Model

The measurement model included four latent variables (school climate, surface acting, deep acting and emotional exhaustion) and 23 observed variables. Fit indices revealed good model fit: χ^2^ (75, N = 703) = 687.70, *p* < 0.001, RMSEA (90% CI) = 0.054 (0.050, 0.059), CFI = 0.93, TLI = 0.92, and SRMR = 0.057. All the factor loadings for each indicator on the latent variables were significant (*p* < 0.001), showing that all the latent factors were well represented by their indicators respectively. In addition, as shown in Table 1, all the latent factors from the measurement model were significantly intercorrelated (*p* < 0.05).

### 4.3. Structural Model

The direct path coefficient from the predictor variable (school climate) to the dependent variable (emotional exhaustion) in the absence of mediators was significant (β = −0.33, *p* < 0.01). Then the results of a partially-mediated model with two mediators (surface acting and deep acting) and a direct path from school climate to emotional exhaustion revealed a good fit to the data: χ^2^ (74, N = 703) = 688.125, *p* < 0.001, RMSEA (90% CI) = 0.054 (0.050, 0.059), CFI = 0.93, TLI = 0.92, and SRMR = 0.057. Moreover, in the partially-mediated model, the direct path coefficient from school climate to emotional exhaustion was significant (β = −0.23, *p* < 0.01), so the partial mediation model was chosen for the interpretation of the effects.

**Figure 1:**
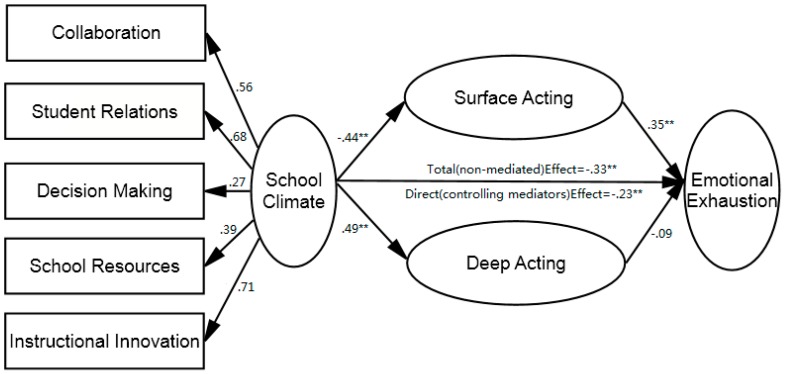
Model of relationship among school climate, emotional labor strategies and emotional exhaustion (*N* = 703). *Note*: parameters presented were standard regression coefficients. ** *p* < 0.01.

As is shown in Figure 1, the school climate negatively predicted surface acting (β = −0.44, *p <* 0.01) and positively predicted deep acting (β = 0.49, *p <* 0.01). Surface acting significantly predicted emotional exhaustion (β = 0.35, *p <* 0.01), while deep acting did not. Further, the total (non-mediated,) and direct (β = −0.29, *p* < 0.01) effects of the school climate on emotional exhaustion were both significant. The total indirect effect of the school climate through the emotional labor strategy was −0.11, with a 95% bootstrap CI of −0.49 to −0.08. Specifically, the indirect effect through surface acting was −0.16, with a 95% CI of −0.56 to −0.28, and the indirect effect through deep acting was not significant and was estimated to be 0.05, with a 95% CI of −0.01 to 0.10.

## 5. Discussion

The purpose of the present study was to examine the relationships between Chinese teachers’ perceptions of the school climate, emotional labor and emotional exhaustion. Several key findings and their implications are discussed below.

### 5.1. The Effects of the School Climate on Emotional Labor

Consistent with our hypothesis, the results revealed that the teachers’ perceptions of the school climate had a negative effect on surface acting and a positive effect on deep acting. This finding confirms the inference that organizational factors may be an antecedent of employees’ emotional labor. Based on the perspective of self-determination, Cossette [38] argued that motivation is a powerful predictor of emotional labor, and previous research has shown that work motivation can be fostered by the social context in which one works. 

However, this finding of the present study is partially inconsistent with the work of Hur, Moon, and Jun, who found that perceived organizational support significantly influences flight attendants’ use of deep acting, but does not influence surface acting [39]. Interestingly, the organization climate (e.g., school climate and organizational support) influenced the teachers’ use of surface acting but not the flight attendants’. The reason for the inconsistency may lie in the different motivational processes for performing emotional labor of the two occupations. The emotional display rules for teachers are not as strict and precise as commercial organizations (e.g., airline companies) [16,40]. This suggests that the flight attendants’ performance of surface acting is influenced more by organizational demand (external motivation) than by inner autonomous need (internal motivation), whereas this opposite situation is true for teachers. Hence, it seems that a supportive organizational environment has a greater affect on inner motivation than on external motivation. This finding extends affective event theory by incorporating the role of motivation into the explanation of the relationship between the organizational environment and affective behavior. Additionally, this finding also enriches the emotional labor literature by considering the school climate to be an antecedent of emotional labor. 

### 5.2. Effects of Emotional Labor on Emotional Exhaustion

The multiple mediation analysis revealed that surface acting was significantly related to emotional exhaustion among Chinese teachers, whereas deep acting was not. These results are consistent with those of a study by Cheung *et al.* [13] that was also conducted among Chinese primary and junior high school teachers. However, the results are inconsistent with those of the Turkish study of Akın *et al.* [14], which found that surface acting among primary school teachers was positively related to emotional exhaustion, whereas deep acting was negatively related to emotional exhaustion. Furthermore, the results of the present study are also inconsistent with those of a study of a sample of Chinese kindergarten teachers [15] that found the same result as Akin *et al.* [14]. One possible reason for this inconsistency may lie in the time span of the teacher-student interactions. In Chinese kindergartens, teachers are often required to take care of the same children from when they enter school until they graduate [41], which entails spending three or four years with the same group of students. However, in most Chinese primary and high schools, teachers typically spend only half a year or one year with the same students. In contrast, primary school teachers in Turkey are required to teach in the first four grades [14]. It seems that with an increasing relationship duration, the effect of deep acting on emotional exhaustion changes from zero to negative. 

According to the ego-depletion theory, deep acting is an effortful emotional regulation process that drains mental resources and leads to burnout to some extent [10]. However, deep acting may benefit the employees’ well-being in two manners. First, because the performance of deep acting involves changes in inner feelings, it may increase employees’ authentic feelings and thus decrease their emotional dissonance [10,42]. Second, according to the social interaction model suggested by Coté [43], increasing positive emotions through deep acting elicits favorable responses from the partner, which in turn positively affect the employees’ well-being. Taken together, these findings indicate that deep acting has both costs and benefits and that the relationship between deep acting and employees’ well-being (e.g., emotional exhaustion) may depend on the balance of the costs and benefits. Specifically, if the costs are greater than the benefits, deep acting is positively related to emotional exhaustion. For example, if a ticket seller makes great effort to understand his customers and display positive emotions from his heart, however, most customers are in a hurry, some are even disrespectful and rude, as a result, the ticket seller’s effort are not appreciated and has no impact on his customers’ behaviors because of the limited time of interaction. In this case, due to the high cost and low benefits, the ticket seller would become more emotional exhausted. In contrast, if the benefits are greater than the costs, deep acting is negatively related to emotional exhaustion. For example, if a teacher always tries hard to understand certain student’s misbehavior and displays positive emotion to her/him, it is likely that the student would feel grateful and behave accordingly, because the long-term interaction could build a sense of trust and understanding between the student and the teacher, So that the teachers’ effort are appreciated by the student and the teacher would experience more rewards for her/his effort and less emotional dissonance as well as more well-being. If the costs and benefits are equal, deep acting is unrelated to emotional exhaustion. For example, due to the relative short-term interaction with students, a teacher who tries hard to understand the misbehaviors of her/his students may not be able to build a high-level trustful relationship with her/his students as in the former case, then it is possible that she/he would experience some rewards and equal amount of costs, in this case, it is impossible to predict her/his emotional exhaustion based on her/his effort in deep acting. According to interpersonal relationship theory [44], the benefits of deep acting increase with increasing relationship duration due to the enhancement of trust; thus, the effect is more likely to be salient in long-term interactions. Further research should explore the moderator role of the relationship duration in the relationship between different emotional labor strategies and employees’ well-being. Although the moderating effect of relationship duration was not the only possible explanation, there may still be other factors that have impact on the relationship between deep acting and emotional exhaustion. Further research should also identify those factors. 

### 5.3. The Mediating Role of Emotional Labor

In addition to a direct effect, the teachers’ perceptions of the school climate also had an indirect effect on emotional exhaustion through surface acting. For this reason, it is likely that teachers who perceive high quality school climates adopt less surface acting when faced with emotional dissonance. Consequently, these teachers experience less emotional exhaustion in subsequent work. These findings further confirmed previous work indicating that teachers’ burnout is significantly influenced by their perception of students’ misbehavior through different coping strategies (e.g., emotional-focused strategies) [45]. The current study also extends previous work by incorporating the role of emotional labor into the relationship between teachers’ perceptions of the school climate and emotional exhaustion [20,21,22]. These findings highlight the importance of teachers’ perceptions of the school climate and emotional labor strategies in inhibiting negative teacher outcomes, such as burnout [20], job dissatisfaction [25,46] and turnover [26]. These findings also provide evidence that could aid in the identification of methods to reduce teacher burnout. For example, teachers’ burnout could be alleviated via the improvement of school climate or the adjustment of emotional labor strategies. Additionally, although the majority of the previous research [12,47,48,49] has conceptualized the organizational environment as a moderator in the relationship between emotional labor and burnout, a growing body of theoretical and empirical research has begun to consider the antecedent effect of the organizational environment on emotional labor [38,39,50]. The present research deepened the previous emotional labor studies by developing a mediation model in which the perceived school climate acted as an antecedent for emotional labor strategy and emotional exhaustion.

### 5.4. Limitations

The current study has some limitations. First, due to the cross-sectional design, the relationships observed in this research were correlational in nature. Future longitudinal research is needed to establish the causalities of the relationships between the variables. Second, single-source bias may have been present in the data collection process of the current study. Mixed methods should be employed when collecting data from various sources in future research; for example, data could be collected from students or teaching records. Third, despite our efforts to ensure the representativeness of the sample, it is possible that the teachers who agreed to participate in the study were special in some manner. For example, it is possible that only the teachers who performed well in school had the opportunity to participate in the training course. Furthermore, because all of the research data were derived from only three provinces in China, the results may be limited.

## 6. Conclusions

In short, this study revealed that the relationship between teachers’ perceptions of the school climate and emotional exhaustion is mediated by emotional labor strategies. Moreover, this study confirmed that the organization factor can be an antecedent for employees’ emotional labor strategies. These results have the following implications for enhancing teachers’ subjective well-being and professional development. First, principals should make an effort to foster a high quality working environment in which teachers will feel a strong sense of duty to devote themselves to their work. Second, it is very important to help teachers better understand how their feelings are triggered and which emotional labor strategies they should adopt when the emotional dissonance arise. 
